# Trajectories of Proactive Health Behaviors Among Chinese Middle-Aged and Older Adults with Multimorbidity: A Cohort Study Using Group-Based Trajectory Modeling

**DOI:** 10.3390/ejihpe16030038

**Published:** 2026-03-06

**Authors:** Jiaxuan Wang, Ziqi Wang, Fan Du, Jiaojiao Lv, Jiulong Kou, Jieting Chen, Mingxia Jing

**Affiliations:** 1 Department of Public Health, Shihezi University School of Medicine, Shihezi 832000, China; wangjiaxuan@stu.shzu.edu.cn (J.W.); wangziqi@stu.shzu.edu.cn (Z.W.); dufan@stu.shzu.edu.cn (F.D.); lvjiaojiao@stu.shzu.edu.cn (J.L.); 20232114063@stu.shzu.edu.cn (J.K.); 2 Department of Humanities and Nursing, Shihezi University School of Medicine, Shihezi 832000, China

**Keywords:** multimorbidity, proactive health behaviors, group-based trajectory modeling, generalized structural equation modeling, COM-B model

## Abstract

(1) Background: Proactive health behaviors are key to reducing their burden and supporting healthy aging. (2) Methods: We analyzed five waves (2011–2020) of CHARLS data from 1343 middle-aged and older adults (≥45 years) with multimorbidity. An entropy weight method was used to create a composite score for proactive health behaviors, and group-based trajectory modeling identified behavioral trajectories. Multivariate logistic regression and Shapley value decomposition assessed determinants and their relative contributions. Generalized structural equation modeling and latent class analysis were applied to estimate direct and indirect effects across the full sample and key multimorbidity subgroups. (3) Results: Two trajectories emerged: a “declining group” (91.44%) and an “improving group” (8.56%). The improving group was more likely to include younger, urban individuals with higher education, retired status, smaller family size, and lower depression levels. Education (40.67%) and depressive symptoms (31.22%) were the strongest determinants of trajectory. Path analysis showed that higher education and retirement indirectly supported sustained proactive health behaviors by reducing depression. The direct and indirect effects varied across subgroups. (4) Conclusion: The proactive health behaviors of middle-aged and elderly patients with multimorbidity exhibit a declining trend. Future health policies and interventions should prioritize mental health.

## 1. Introduction

The rapid aging of the Chinese population has led to a substantial increase in the burden of chronic non-communicable diseases, among which multimorbidity—the co-occurrence of two or more chronic conditions in an individual—represents the most significant challenge to the healthcare system (X. Chen et al., 2022; Mezzich & Salloum, 2008). Multimorbidity is not simply the aggregation of multiple diseases but a complex clinical state characterized by the interplay of symptoms, polypharmacy, functional decline, and psychological comorbidities, rendering traditional disease-specific treatment models inadequate for effective management (World Health Organization, 2008). Older adults and middle-aged individuals constitute the primary populations affected by multimorbidity (Chowdhury et al., 2023). Global evidence indicates that over half of people in these age groups are living with two or more concurrent chronic conditions (Ni et al., 2023). In China, the prevalence of multimorbidity among middle-aged and older adults exceeds 57%, rising to more than 75% among those aged 60 years and above (Fan et al., 2022; World Health Organization, 2013). Compared to single chronic diseases, multimorbidity markedly increases the risks of disability, hospitalization, and mortality, while contributing to an exponential rise in healthcare expenditures, thereby accounting for a major component of the overall disease burden in the country (H. Liu et al., 2022; Ma et al., 2020). In this context, the World Health Organization highlights that up to 70% of the global disease burden could be prevented or delayed through enhanced health-related behaviors (World Health Organization, 2013). A substantial body of research has demonstrated that positive health behaviors can significantly assist individuals with multiple chronic conditions in better managing their diseases (Zhang et al., 2024), reducing reliance on pharmacological treatments (Levin & Hanson, 2020), improving psychological well-being (Li et al., 2025), and preserving physical functioning (Skou et al., 2025), thereby markedly enhancing overall quality of life. To shift away from a reactive model of health management, China has introduced the “proactive health” strategy, which positions individuals as the primary stewards of their own health (Central Committee of the Communist Party of China & The State Council of the People’s Republic of China, 2016). Within this framework, proactive health behaviors are defined as self-initiated daily practices and lifestyle modifications adopted by individuals to promote health, prevent illness, and manage chronic conditions effectively (J. Liu et al., 2022), serving as a critical mechanism for translating the concept of “proactive health” into practical action. Consequently, investigating the dynamic patterns and determinants of proactive health behaviors among older adults with multimorbidity is of pressing importance for the development of targeted, effective, and sustainable health interventions.

Although the importance of proactive health behaviors is increasingly acknowledged, existing research in this domain exhibits several notable limitations. First, the majority of studies have concentrated on patients with single chronic conditions and have primarily employed cross-sectional designs (Wu, 2025; Zhu et al., 2025). This static approach fails to capture the dynamic development of proactive health behaviors over time and neglects potential heterogeneity in behavioral trajectories across different patient subgroups. Second, although various determinants—such as sociodemographic characteristics, mental health, and social environment—have been identified (L. Chen et al., 2022; Lee & Jeon, 2025), analyses largely remain at the level of associative exploration, lacking in-depth mechanistic investigation. In particular, among middle-aged and older adults with multiple chronic conditions, the variability in longitudinal patterns of proactive health behaviors has been overlooked, and determinants have not been comprehensively examined from multidimensional perspectives encompassing capability, opportunity, and motivation. Furthermore, current studies have neither quantified the relative contribution of each determinant nor systematically analyzed the pathways through which these factors influence heterogeneous behavioral trajectories (L. Chen et al., 2022; Lee & Jeon, 2025; Wu, 2025; Zhu et al., 2025).

To fill these research gaps, comprehensively characterize the long-term evolution patterns of proactive health behaviors among middle-aged and elderly patients with multimorbidity in China, and contribute to the achievement of the strategic goal of healthy aging, this study utilizes five waves of longitudinal data from the China Health and Retirement Longitudinal Study (CHARLS) to explore the following three questions: (1) Do the proactive health behaviors of middle-aged and elderly patients with multimorbidity in China exhibit heterogeneous development trajectories over time? (2) Under the COM-B behavior framework (Michie et al., 2011), are there differences in the relative contributions of general demographic characteristics, capabilities, opportunities, and motivation factors to the trajectories of proactive health behaviors, and through what direct and indirect pathways do these factors exert their effects? (3) Do the above-mentioned influence pathways vary among different multimorbidity subgroups?

To address these questions, based on the COM-B model, this study employs the group-based trajectory modeling (GBTM) (Nguena Nguefack et al., 2020) and path analysis methods and proposes four hypotheses: (H1) There are heterogeneous development trajectories of proactive health behaviors; (H2) There are differences in the relative contributions of general demographic characteristics, capabilities, opportunities, and motivation factors to the trajectories of proactive health behaviors; (H3) Capabilities and opportunities not only directly affect the trajectories of proactive health behaviors but also have indirect effects through motivation factors; and (H4) The effect pathways are heterogeneous among different multimorbidity subgroups.

## 2. Materials and Methods

### 2.1. Data Sources

The data for this study were sourced from the publicly available dataset of the China Health and Retirement Longitudinal Study (CHARLS). The analytical sample included middle-aged and elderly individuals aged 45 years and above at baseline who reported having multiple chronic diseases and who participated in all four follow-up waves conducted in 2013 (Wave 2), 2015 (Wave 3), 2018 (Wave 4), and 2020 (Wave 5). Participants with missing key health behavior variables or potential influencing factor variables in either the baseline or follow-up data were excluded. A final sample of 1343 individuals was retained for analysis (Supplementary Figure S1). The CHARLS dataset encompasses information on 14 chronic conditions, including hypertension, diabetes, cancer, chronic lung diseases, heart disease, stroke, mental illness, arthritis, dyslipidemia, liver disease, kidney disease, stomach disease, asthma and memory disorders.

### 2.2. Proactive Health Behaviors

Proactive health behaviors refer to the systematic daily practices that individuals voluntarily adopt based on the concept of proactive health, aiming to enhance health levels, prevent diseases, and delay the progression of chronic diseases. The concept of proactive health not only emphasizes physical health but also attaches importance to the integrity of social functions. Therefore, unlike traditional health behaviors that only focus on physiological health habits (such as exercise or quitting smoking), the evaluation dimensions of proactive health behaviors are more diverse, covering not only healthy lifestyles but also proactive health practices such as social participation (J. Liu et al., 2022). Based on previous studies (Zhou et al., 2025), this paper evaluates the level of proactive health behaviors from three dimensions, health exercise, health habits, and health social interaction, and combines the entropy weight method for a comprehensive assessment (Table 1 and Supplementary Table S1).

### 2.3. Proactive Health Behavior Score

Proactive health behaviors encompass three dimensions—health exercise, health habits, and health social interaction—assessed through 21 evaluation indicators (Table 1). To mitigate the bias associated with subjective weight assignment, this study employs the entropy weight method to objectively assign weights based on the degree of data variation across each indicator, thereby constructing a comprehensive score for proactive health behaviors.

(1) Data Standardization: For the evaluation of proactive health behaviors, with n samples and m indicators, the value of the j indicator for the i sample is denoted by $x_{ij}$ (i = 1, 2, …, n; j = 1, 2, …, m). Standardization is applied separately to positive and negative indicators based on their attributes to eliminate incomparability among the data.

(2) Compute the proportion of the i sample value under the j indicator:

$$p_{\mathrm{ij}}=x_{\mathrm{ij}}/\sum_{i=1}^{n}x_{\mathrm{ij}}$$

$p_{ij}$ represents the proportion of the indicator;

(3) Calculate the entropy value for the j indicator:

$$e_{j}\;=\;-k\sum_{i=1}^{n}p_{\mathrm{ij}}\ln p_{\mathrm{ij}}$$

$k=\frac{1}{\ln\left(n\right)}>0$, $e_{j}\geq 0$;

(4) Calculate the redundancy of information entropy:

$$d_{j}\;=1-e_{j}$$

(5) Calculate the weights of each indicator:

$$w_{j}=d_{j}/\sum_{i=1}^{m}d_{j}$$

(6) Calculate the comprehensive score of proactive health behaviors:

$$s_{i}=\sum_{i=1}^{m}w_{j}x_{\mathrm{ij}}$$

In this study, $s_{i}$ represents the proactive health behavior score of the i sample and $x_{\mathrm{ij}}$ is the standardized data.

### 2.4. Determinants

**Figure 1:**
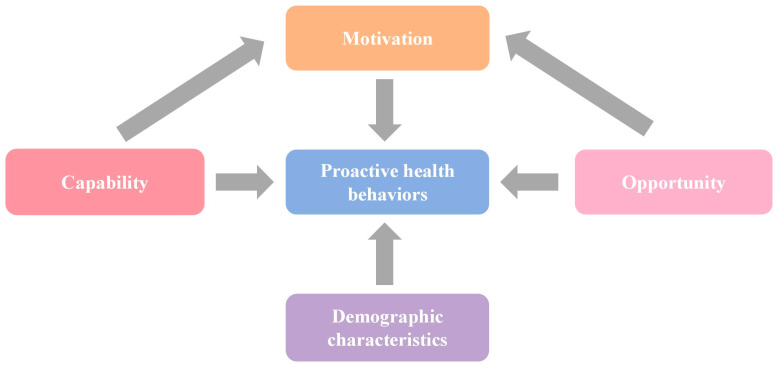
Determinants and pathways of proactive health behaviors.

Combined with the COM-B model, the factors affecting proactive health behaviors were divided into general demographic characteristics, ability factors, motivation factors and opportunity factors (Zhou et al., 2025). The COM-B model elaborates the occurrence mechanism and influencing factors of behavior change around the three core elements of capability, opportunity and motivation. At the same time, the model points out that ability factors and opportunity factors can indirectly affect the occurrence and change in behavior through motivation factors (Michie et al., 2011) (Figure 1). For general demographic characteristics, age (expressed by median and interquartile range), gender (male, female), marital status (married, other), and family size (expressed by median and interquartile range) were selected for consideration of data availability. Ability factors included educational level (primary school or below, middle school, high school or above), instrumental activities of daily living (IADL, no difficulty, some difficulty, very difficulty), and activities of daily living (ADL, no difficulty, some difficulty, very difficult). Chance factors included residence (urban, rural), retirement (yes, no), and medical insurance (yes, no); the motivational factor was depression score (expressed as median and interquartile range).

First, the marital status category “other” encompasses four distinct subcategories: separated, divorced, widowed, and never married. Second, family size is defined as the total number of individuals residing together in a household, including the respondent, spouse, all cohabiting family members, and those temporarily absent but not yet financially independent. Depressive symptoms were assessed using the Center for Epidemiologic Studies Depression Scale—Short Form (CES-D-10), which consists of 10 items, each rated on a 4-point scale ranging from 0 to 3. The total score, derived by summing responses across all items, ranges from 0 to 30, with higher scores indicating greater severity of depressive symptoms (H. Chen & Mui, 2014; R. Chen et al., 2005). Fourth, instrumental activities of daily living (IADL) and basic activities of daily living (ADL) were evaluated using standardized scales. IADL was measured using a five-item scale assessing the ability to manage finances, shop, prepare meals, conduct business, and take medication. ADL was assessed using a six-item scale evaluating dressing, bathing, eating, transferring (getting in and out of bed), using a toilet, and bowel and bladder control. Each item on both scales offered four response options: (1) not difficult at all, (2) somewhat difficult but manageable without assistance, (3) somewhat difficult and requiring help, and (4) unable to complete. For scoring purposes, responses indicating any difficulty or inability were coded as 1, while no difficulty was coded as 0. Consequently, IADL scores ranged from 0 to 5 and ADL scores from 0 to 6. In this study, both IADL and ADL statuses were categorized into three levels: no difficulties (score = 0), some difficulties (score = 1–2), and severe difficulties (score ≥ 3) (You et al., 2023).

### 2.5. Statistical Analysis

Stata18.0 was used to calculate the comprehensive score of proactive health behaviors, construct the proactive health behavior trajectory, and identify the importance of determinants and action paths of proactive health behavior trajectories. The entropy weight method was used to calculate the weight of each indicator of proactive health behaviors (Table 1). The standardized value of each index and the weight of each index were multiplied and added to calculate the comprehensive score of proactive health behaviors. For the convenience of observation, the calculated proactive health behavior score was expanded by 10 times, and the value was divided into 0 to 10 points; the higher the score, the better. The group-based trajectory model (GBTM) was used to construct the proactive health behavior trajectory, and AIC, BIC, aBIC, entropy, trajectory sample rate and average posterior probabilities (Avepp) were selected to evaluate the fitting degree of the model. The lower the AIC, BIC and aBIC values, the better the model fit; the higher the entropy values, the higher the classification accuracy; the sample rate of the trajectories is not less than 5% (Nguena Nguefack et al., 2020). Multivariate logistic regression analysis was used to identify the determinants of the participants’ trajectories. Based on the results of multivariate logistic regression analysis, Shapley value decomposition was used to evaluate the contribution of each determinants. Furthermore, generalized structural equation modeling (GSEM) was used to explore the direct and indirect effects of determinants on trajectories (Rabe-Hesketh et al., 2004). Using Mplus8, latent class analysis (LCA) was used to identify the main multimorbidity combinations of the population, and the path analysis of different comorbidity combinations was further explored (Sinha et al., 2021). The median and quartile were used for statistical description, and the rate and constituent ratio of classification and registration data were used for statistical description. Chi-square tests or rank sum tests were used for comparison between groups. Statistical analysis was performed using a two-sided test and *p* < 0.05 was considered statistically significant.

## 3. Results

### 3.1. Baseline Characteristics of Chinese Middle-Aged and Elderly Patients with Multimorbidity

Among 1343 participants, the median age was 59 years; 38.57% were male, 88.76% were married, 63.14% resided in rural areas, and 72.00% had attained primary education or lower. A small proportion of participants were retired (11.99%), whereas nearly all were covered by health insurance (96.35%). The median values for household size, depression score, and number of comorbidities were 3, 10, and 3, respectively. Additionally, 75.35% of participants reported no difficulties in instrumental activities of daily living or basic activities of daily living (Supplementary Table S2).

### 3.2. Trajectory of Proactive Health Behaviors Among Chinese Middle-Aged and Elderly Patients with Multimorbidity

**Figure 2:**
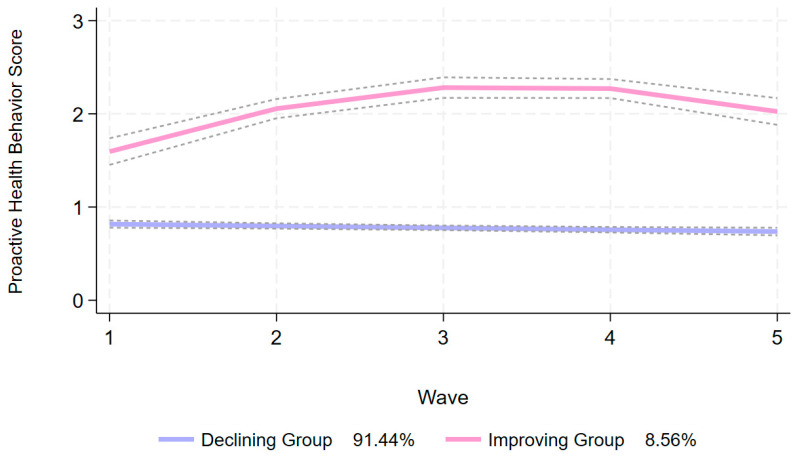
Proactive health behavior trajectory of middle-aged and elderly patients with multimorbidity.

Following a comprehensive evaluation of AIC (−6862.25), BIC (−6880.09), aBIC (−6880.46), entropy values (0.946), minimum class proportions (>5%), and Avepp (>0.7), the optimal CBTM solution was determined to consist of two latent trajectories (Supplementary Table S3). Group 1 exhibited a gradual decline in proactive health behavior score over time (intercept = 0.806, linear = −0.023), and was thus labeled the “declining group” (*n* = 1228, 91.44%). Group 2 demonstrated consistently high levels of proactive health behaviors with a steady upward trend over time (intercept = 0.896, linear = 0.816, quadratic = −0.118), and was accordingly classified as the “improving group” (*n* = 115, 8.56%) (Figure 2).

### 3.3. Determinants of Proactive Health Behavior Trajectories in Chinese Middle-Aged and Elderly Patients with Multimorbidity

**Figure 3:**
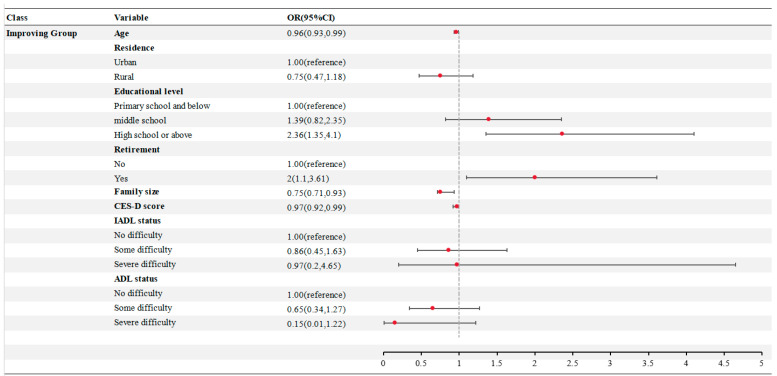
Multivariate logistic regression analysis of proactive health behavior trajectory in middle-aged and elderly patients with multimorbidity.

There were statistically significant differences in age, residence, education level, retirement, family size, CES-D score, IADL, and ADL between the two groups (*p* < 0.05). No statistically significant differences were observed in gender, marital status, medical insurance coverage, or number of multimorbidities (*p* > 0.05) (Supplementary Table S2). In the multivariate logistic regression analysis, the GBTM group was designated as the dependent variable, while the independent variables included factors that showed statistically significant differences in the univariate analysis, with the declining group serving as the reference category. Age, education level, retirement status, family size, and mental health emerged as significant determinants influencing the proactive health behavior trajectory among middle-aged and elderly patients with multimorbidity (Figure 3). Specifically, older age, urban residency, retired status, education level at or above senior high school, smaller family size, and lower depression scores were associated with a higher likelihood of belonging to the stable-increase group in terms of health behavior scores.

### 3.4. Contributions of Determinants to Proactive Health Behavior Trajectories Among Chinese Middle-Aged and Elderly Patients with Multimorbidity

Based on the results of multivariate logistic regression analysis, the Shapley value decomposition method was employed to assess the relative contributions of determinants across different trajectory groups. In the declining group, CES-D score exhibited the highest contribution (37.12%), followed by education level (26.61%), age (25.56%), retirement (6.60%), and family size (4.11%). In contrast, within the improving group, education level was the primary contributing factor (40.67%), followed by depression score (31.22%), retirement status (17.60%), family size (5.17%), and age (4.80%) (Supplementary Figure S2).

### 3.5. Path Analysis of Determinants of Proactive Health Behavior Trajectory Among Chinese Middle-Aged and Elderly Patients with Multimorbidity

**Figure 4:**
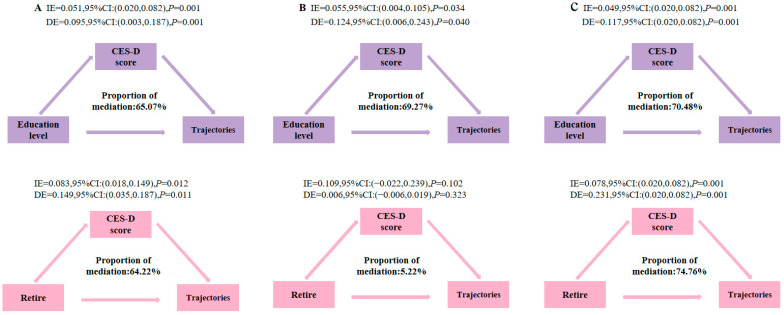
Indirect effects of determinants of proactive health behavior trajectories among Chinese middle-aged and elderly patients with multimorbidity. Note: (**A**) total, (**B**) arthritis–stomach and (**C**) cardiometabolic–arthritis. IE: direct effect, DE: indirect effect, total effect: direct effect + indirect effect; proportion of mediation = indirect effect/total effect × 100%.

The path analysis results indicated that age, family size, and depression exerted significant direct negative effects on trajectories, whereas retirement and education level demonstrated significant direct positive effects. Meanwhile, both retirement and education level exhibited indirect effects on trajectories through CES-D score. Specifically, education level had a significant positive indirect effect on trajectories via CES-D score, with a total indirect effect of 0.095, representing 65.07% of the total effect. Similarly, retirement showed a significant positive indirect effect on trajectories through depression score, yielding an indirect effect of 0.149, which accounted for 35.77% of its total effect (Table 2 and Figure 4). Based on the indicators such as AIC (14,5054.31), BIC (14,439.31), aBIC (14,204.25), entropy value (0.681), LMR (*p* = 0.003) and BLRT (*p* < 0.001) of LCA, the five-category model had the best fit (Supplementary Table S4). Combined with the conditional probability distribution of the five categories and the clinical literature on comorbidity patterns, this study ultimately identified two main comorbidity patterns: arthritis–stomach (*n* = 500, 37.23%) (Baraliakos et al., 2023) and cardiometabolic–arthritis (*n* = 843, 62.77%) (Drosos et al., 2024) (Supplementary Table S5). Subgroup analyses revealed that, within the arthritis–gastric disease group, age, family size, and depression had direct negative effects on trajectories, while education level had a direct positive effect. Education level also influenced trajectories indirectly through depression score, demonstrating a significant positive indirect effect of 0.124, constituting 69.27% of the total effect. In the cardiometabolic–arthritis group, education level similarly exerted a significant positive indirect effect on trajectories through CES-D score (indirect effect = 0.117, 70.48% of total effect). Retirement was associated with a significant positive indirect effect on trajectories through CES-D score in this group, with an indirect effect of 0.231, accounting for 74.76% of the total effect (Table 2 and Figure 4).

## 4. Discussion

### 4.1. Trajectory of Proactive Health Behaviors

The study found that there were two types of proactive health behavior trajectories of middle-aged and elderly Chinese patients with multimorbidity: the declining group (accounting for 91.44%) and the improving group (accounting for 8.56%), which was not consistent with the conclusion that the health behavior of middle-aged and elderly people is generally stable (Feng, 2021; Luo et al., 2020). As people age and accumulate chronic diseases, the worsening of physical symptoms, the accelerated decline of physical skills, and the complex care needs of patients with multimorbidity may gradually limit their ability to maintain and improve proactive health behaviors (Cesare et al., 2024; Langenberg et al., 2023; Skou et al., 2025). The score of proactive health behavior in the improving group continued to increase from 2011 to 2018. This coincided with the release of the National Chronic Disease Prevention and Control Plan (2012–2015), the Outline of the Healthy China 2030 Plan, the 13th Five-Year Plan for Health and Wellness (Central Committee of the Communist Party of China & The State Council of the People’s Republic of China, 2016; Guo, 2012; Xiao, 2017), and other documents at the national level, as well as the improvement of the service capacity of primary medical and health institutions. This suggests that changes in the macro policy environment may improve health behaviors by improving health accessibility and health literacy (Deng, 2022; Palmer et al., 2024). Furthermore, the turning point in the improving group between 2018 and 2020 might be attributed to the impact of the COVID-19 pandemic in 2019, which restricted the participation of middle-aged and elderly people in outdoor activities. The above findings suggest that it is possible to change the active health behaviors of middle-aged and elderly patients with multimorbidity. The characteristics of education level, residence and mental health and the important role of health policy implementation should be fully considered, so as to provide strong evidence for the design of proactive health behavior intervention for middle-aged and elderly patients with multimorbidity.

### 4.2. Determinants of Proactive Health Behavior Trajectories

The multivariate logistic regression model showed that age, education level, retirement, family size and depression were independent determinants to distinguish the two trajectories. In terms of general population characteristics, increasing age and increasing family size reduce the probability of individuals entering the improving group. The reasons for this may be the decline of physical function accompanied by aging, the accumulation of multimorbidity, and age discrimination, which weakens the ability to maintain behavior (Bloomberg et al., 2024). At the same time, small-scale families may not only allow people to devote more time and energy to health due to less intergenerational conflict and less care burden, but also facilitate indoor exercise and diet management due to improved living conditions (Song & Chen, 2025; Wang, 2024). In terms of ability, the probability of entering improving group with an education level of senior high school or above was 2.36 times that of primary school or below. Education level is positively correlated with higher health literacy, information screening ability and health self-efficacy (Baker et al., 1998; Sudore et al., 2006); disease management actively adopts doctors’ advice, health management compliance is high, and proactive health behaviors are maintained and improved. At the opportunity level, retirees are more likely to maintain and promote proactive health behaviors, which may be due to the increased time autonomy after retirement, which is conducive to regular exercise, social activities and comorbidity self-management (Barnett et al., 2012; Pronk et al., 2004). At the motivational level, depression score was a risk factor for proactive health behavior trajectory. Depression hinders the initiation and maintenance of healthy behaviors by reducing motivation, increasing fatigue and weakening executive function (Kraft et al., 2023; Snyder, 2013). At the same time, depressed patients are more likely to experience social withdrawal and helplessness and further reduce social and physical activities (Elmer & Stadtfeld, 2020; Steger & Kashdan, 2009). The above results suggest that health education interventions should focus on low-education groups, pay attention to mental health intervention, bridge the difference between education levels by simplifying information, peer education and community classrooms, and carry out community psychological screening, peer support and digital psychological interventions to reduce the burden of depression, improve the level of proactive health behaviors, and maintain the effect of health intervention.

### 4.3. Contributions of Determinants to Proactive Health Behavior Trajectories

The Shapley value decomposition further quantified the relative contributions of each independent determinant to trajectories. In the declining group, depression emerged as the primary inhibiting factor, followed by educational level. In contrast, within the improving group, educational level contributed the most, followed by depression, while age exhibited a significantly greater contribution in the continuously declining group compared to the improving group. These findings align with the preceding analysis and reinforce the critical role of mental health status and educational attainment in health behavior interventions among middle-aged and elderly patients with multimorbidity, which is consistent with prior research (World Health Organization, 2007; Sardareh et al., 2024). Considering the older demographic profile of the declining group, it is likely that declining physical health with advancing age constrains their engagement in health-promoting behaviors. Evidence suggests that, as age increases, older adults may experience negative emotions due to reduced work capacity, deteriorating physical function, and diminished social roles, yet they often internalize or stigmatize depression, thereby concealing their psychological distress and delaying help-seeking behaviors (Mackenzie et al., 2019; Polacsek et al., 2019). Therefore, primary healthcare institutions should implement regular psychological screening and mental health education programs to assist middle-aged and elderly individuals in developing a scientifically informed understanding of mental health. During health education initiatives, a peer-support model could be adopted by forming mixed-education groups, wherein individuals with higher educational attainment serve as peer supporters to facilitate the comprehension and adoption of health information among those with lower educational levels. Furthermore, although retirement status and family size were statistically significant, their effect sizes were relatively small, indicating that their influence may be mediated through interactions with other variables.

### 4.4. Path Analysis of Determinants of Proactive Health Behavior Trajectories

The results of the overall path analysis indicated that age, family size, and depression exerted significant negative effects on trajectories. Retirement and education level not only positively influenced proactive health behavior trajectories directly but also demonstrated indirect positive effects through depression, which served as a mediating factor. This might be attributed to the fact that higher education levels enhance health literacy and cognitive capacity, thereby reducing the sense of helplessness and anxiety associated with multimorbidity, and thus preventing the occurrence and development of depressive symptoms. Similarly, retirement may improve emotional well-being by alleviating work-related stress, increasing temporal autonomy, and facilitating adjustments in daily routines (Barnett et al., 2012; Elmer & Stadtfeld, 2020; Kraft et al., 2023; Snyder, 2013). Together, these factors may alleviate the psychological burden experienced by individuals with multimorbidity, thereby enhancing their motivation and self-management capabilities in adopting proactive health behaviors. These findings further underscore the considerable potential of mental health interventions in promoting proactive engagement in health-related behaviors.

The results of the subgroup analysis indicated that although different multimorbidity combinations did not alter the overall direction of the effects for each pathway, variations were observed in terms of statistical significance and effect size. Specifically, the direct paths associated with age and retirement failed to reach statistical significance in the arthritis–stomach group, whereas they remained statistically significant in the cardiometabolic–arthritis group. This difference may be attributed to the relatively rapid onset but reversible nature of osteoarticular and gastrointestinal symptoms, which can improve shortly after removal of triggering factors and through standardized interventions (Baraliakos et al., 2023; Shahid et al., 2025), coupled with a low disability rate (Collaborators, 2024). As a result, individuals with arthritis and gastropathy may be less sensitive to age-related discrimination and perceived value loss associated with aging and retirement. In contrast, patients with cardiometabolic disease and arthritis in China face a higher risk of acute complications (Collaborators, 2024) and greater challenges regarding treatment adherence (Drosos et al., 2024). They are therefore more responsive to institutional health support mechanisms linked to aging and retirement—such as routine health examinations for retirees, increased outpatient reimbursement for chronic conditions, and expanded primary public health services—explaining the continued significance of these pathways. Secondly, with respect to the proportion of indirect effects, the education level exhibited an indirect effect of 69.27% in the arthritis–stomach group and 70.48% in the cardiometabolic–arthritis group, both of which exceed the overall average. The results suggest that the positive impact of educational level on health behaviors in specific multimorbidity contexts may be partially achieved through the alleviation of depression. This phenomenon may be attributed to the enhanced capacity of individuals with higher educational attainment to access, interpret, and apply health-related information, thereby reducing the complexity of health decision-making (Geurts et al., 2022; Singh et al., 2025). This cognitive advantage is subsequently internalized into improved self-management skills, enabling patients to regulate their emotions more effectively and engage in proactive health-promoting behaviors. It is noteworthy that the indirect effect of retirement in the cardiometabolic–arthritis group increased substantially to 74.76%, markedly higher than the 35.77% observed in the overall sample, whereas this effect was not statistically significant in the arthritis–stomach group. The findings indicate that among patients with cardiometabolic–arthritis, time flexibility and institutional support after retirement may promote the implementation of proactive health behaviors by alleviating depression. In contrast, for the relatively lower-burden arthritis–stomach subgroup, the beneficial impact of retirement on depression was attenuated, resulting in a non-significant indirect pathway. Taken together, the path and subgroup analyses suggest that future health policies and interventions promoting proactive health management should adopt a dual-pronged approach: integrating mental health support as a central linkage, while advancing educational empowerment and optimizing retirement-related health systems to effectively improve health behavior outcomes among middle-aged and older adults with multimorbidity.

### 4.5. Strengths and Limitations

This study represents the first investigation into the developmental trajectories of proactive health behaviors among middle-aged and elderly patients with multimorbidity in China. An innovative methodological approach combining GBTM and GSEM, integrated within the COM-B behavioral framework, was employed to comprehensively examine the longitudinal patterns and determinants of proactive health behaviors in this population. In contrast to conventional cross-sectional designs, this longitudinal analysis enables a more nuanced understanding of the dynamic nature of health behaviors, thereby providing a robust scientific foundation for the development of targeted health interventions. Furthermore, the entropy weight method was applied to compute a composite health behavior score, allowing for an objective integration of multiple behavioral indicators and mitigating the limitations associated with single-metric assessments. Finally, this study, based on a large-scale, nationally representative cohort of middle-aged and elderly people in China, reveals that mental health and educational factors are the key drivers of active health behaviors among patients with multimorbidity. This finding provides important empirical evidence for formulating precise health promotion policies and intervention strategies for middle-aged and elderly people in China.

Several limitations should be acknowledged. First, all data were self-reported, which may introduce information bias. Second, the absence of laboratory and auxiliary test data may have resulted in the omission of key objective biomarkers related to disease status or health behaviors. Third, GSEM lacks mature fit indices, making it difficult to absolutely determine model fit. The results of path analysis are exploratory evidence, and the potential mechanisms it reveals provide hypothesis directions for subsequent research. In future studies, more rigorous methodological approaches should be adopted to verify the causal inference validity. Finally, the sample was restricted to participants from China, which may limit the external validity of the findings when applied to other cultural or national populations.

## 5. Conclusions

There is considerable heterogeneity in the developmental trajectories of proactive health behaviors among middle-aged and elderly individuals with multimorbidity in China, with educational attainment and depression severity emerging as key influencing factors. Therefore, future health policy formulation and intervention measures aimed at promoting proactive health behaviors should give priority to mental health and the educational level of patients with multimorbidity.

## Figures and Tables

**Table 1:** Proactive health behavior indicators and the weight of each index.

Dimension	Primary Indicator	Secondary Indicator	Direction	Weight
Health exercise	Active exercise	Mild activities	+	1.43
		Moderate activity	+	4.33
		Vigorous activity	+	0.48
Health habits	Quit smoking and drinking	Smoking	-	1.39
		Drinking	-	2.59
	Consistent schedule	Duration of nighttime sleep	+	0.44
		Afternoon nap	+	0.63
Health social interaction	Social participation	Interacted with friends	+	2.28
		Activity frequency	+	2.49
		Played Mahjong, played chess, played cards, or went to community club	+	3.73
		Activity frequency	+	3.93
		Provided help to family, friends, or neighbors who do not live with you	+	4.80
		Activity frequency	+	4.98
		Went to a sport, social, or other kind of club	+	5.78
		Activity frequency	+	5.90
		Took part in a community-related organization	+	8.43
		Activity frequency	+	8.55
		Did voluntary or charity work	+	7.67
		Activity frequency	+	7.77
		Attended an educational or training course	+	11.36
		Activity frequency	+	11.04

Note: + = positive indicator; - = negative indicator; activity frequency: each activity frequency corresponds to the activity frequency of the previous cell.

**Table 2:** Direct effects of determinants of proactive health behavior trajectories among Chinese middle-aged and elderly patients with multimorbidity.

	Total		Arthritis–Stomach		Cardiometabolic–Arthritis	
	Coefficient of effect	*p* value	Coefficient of effect	*p* value	Coefficient of effect	*p* value
**Age–Trajectories**	−0.234 (−0.037, 0.010)	0.001	−0.015 (−0.036, 0.005)	0.145	−0.029 (−0.047, −0.011)	0.001
**Family size–Trajectories**	−0.024 (−0.037, −0.010)	0.001	−0.023 (−0.041, −0.004)	0.015	−0.026 (−0.045, −0.001)	0.009
**CES-D score–Trajectories**	−0.026 (−0.039, 0.012)	<0.001	−0.032 (−0.055, −0.008)	0.008	−0.023 (−0.039,−0.006)	0.008
**Educational level–Trajectories**	0.051 (0.020, 0.082)	0.001	0.055 (0.004, 0.105)	0.034	0.049 (0.010, 0.088)	0.015
**Retirement–Trajectories**	0.083 (0.018, 0.149)	0.012	0.109 (−0.022, 0.239)	0.102	0.078 (0.002, 0.155)	0.045
**Educational level–CES-D score**	−0.231 (0.313, −0.149)	<0.001	−0.203 (−0.341, −0.066)	0.004	−0.233 (−0.335, −0.130)	<0.001
**Retirement–CES-D score**	−0.405 (−0.566, −0.243)	<0.001	−0.196 (−0.540, 0.148)	0.263	−0.435 (−0.620, −0.249)	<0.001

Note: total: *n* = 1343, 100.00%; arthritis–stomach: *n* = 500, 37.23%; cardiometabolic–arthritis: *n* = 843, 62.77%.

## Data Availability

The datasets analyzed during the current study are available in the CHARLS repository at https://charls.pku.edu.cn/ (accessed on 11 November 2023).

## Supplementary Materials

The following supporting information can be downloaded at https://www.mdpi.com/article/10.3390/ejihpe16030038/s1. Supplementary Figure S1: Study design; Supplementary Figure S2: Based on Shapley value decomposition, the contribution of multiple factors of proactive health behavior trajectory in Chinese middle-aged and elderly patients with multimorbidity; Supplementary Table S1: Proactive health behavior indicator Description and the assignment of each indicator; Supplementary Table S2: Baseline characteristics of the study participants; Supplementary Table S3: The group-based trajectory model index fitting; Supplementary Table S4: Latent class analysis model fitting index; Supplementary Table S5: Probability distribution of five potential categories.

## Abbreviations

The following abbreviations are used in this manuscript:

CHARLS	China Health and Retirement Longitudinal Study
GBTM	Group-Based Trajectory Model
GSEM	Generalized Structural Equation Modeling
LCA	Latent Class Analysis
IADL	Instrumental Activities of Daily Living
ADL	Activities of Daily Living
