# Supplementary material for: Trajectories of Proactive Health Behaviors Among Chinese Middle-Aged and Older Adults with Multimorbidity: A Cohort Study Using Group-Based Trajectory Modeling
Source: Eur J Investig Health Psychol Educ. 2026 Mar 6;16(3):38. doi: 10.3390/ejihpe16030038 (PMC13026065; doi:10.3390/ejihpe16030038)
Supplement: Supplementary file 1 [file ejihpe-16-00038-s001.zip › ejihpe-4135889-supplementary.pdf]

Supplementary Material

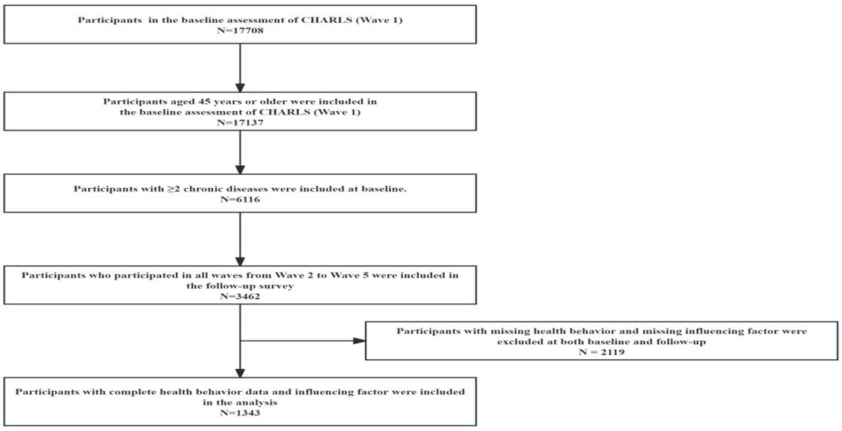

Supplementary Figure S1 Study design

Supplementary Table S1 Proactive health behavior indicator Description and the assignment of eac h indicator

Indicator Description	Assignment
Do you usually take mild activity for at least 10 minutes every week?(1= No, 2= Yes)	1= No, 2= Yes
Do you usually take Moderate activity for at least 10 minutes every week?(1= No, 2= Yes)	1= No, 2= Yes
Do you usually take Vigorous activity for at least 10 minutes every week?(1= Yes, 2= No)	1= No, 2= Yes
Do you currently smoke?(1= Yes, 2= No)	1= No, 2= Yes
Are you currently consuming alcohol?(1= Yes, 2= No)	1= No, 2= Yes
adequate and restful sleep	1 = Insufficient / Excessive sleep time 2 = Good s leep time (6 - 9 hours)
A good habit of taking a nap	1= no naps/long naps 2= good naps (10-30)
Did you do this activity last month?(1= Yes, 2= N o)	1= No, 2= Yes
Activity frequency	0= no to the previous question, 1= not often 2= al most every week 3= almost every day
Did you do this activity last month?(1= Yes, 2= N o)	1= No, 2= Yes
Activity frequency	0= no to the previous question, 1= not often 2= al most every week 3= almost every day
Did you do this activity last month?	1= No, 2= Yes
Activity frequency	0= no to the previous question, 1= not often 2= al most every week 3= almost every day
Did you do this activity last month?	1= No, 2= Yes

Supplementary Table S2 Baseline characteristics of the study participants

Variable	Total	Declining Group	Improving Group	χ^2/Z	*P* value
**Age**	59(53,65)	59(53,65)	57(51,63)	2.747	0.006
**Gender**				0.074	0.786
Male	518(38.57)	475(38.68)	43(37.39)		
Female	825(61.43)	754(61.23)	72(62.61)		
**Marital status**				3.511	0.061
Married	1192(88.76)	1096(89.25)	19(16.52)		
Other	151(11.24)	132(10.75)	96(83.48)		
**Residence**				19.089	＜0.001
Urban	498(36.86)	431(35.09)	63(54.78)		
Rural	848(63.14)	797(64.91)	51(45.22)		
**Educational level**				47.806	＜0.001
primary school or below	967(72.00)	912(74.26)	55(47.82)		
middle school	237(17.65)	208(16.93)	29(25.22)		
high school or above	139(10.35)	108(8.81)	31(26.96)		
**Retirement**				26.708	＜0.001
Yes	161(11.99)	130(10.59)	84(73.04)		
No	1182(88.01)	1098(89.41)	31(26.96)		
**Medical insurance**				0.010	0.919
Yes	1249(96.35)	1183(96.34)	111(96.52)		
No	49(3.65)	45(3.66)	4(3.48)		
**Family size**	3(3,5)	3(2,5)	3(2,4)	2.663	＜0.001
**CES-D score**	10(5,15)	10(6,15)	7(3,11)	4.801	＜0.001
**IADL status**				17.620	＜0.001
No difficulty	977(75.35)	875(71.25)	102(88.70)		
Some difficulty	253(18.84)	241(19.63)	12(10.43)		
Very difficulty	113(8.41)	112(9.12)	1(0.87)		
**IADL status**				9.8684	0.007
No difficulty	1012(75.35)	912(74.27)	100(86.96)		
Some difficulty	244(18.17)	231(18.81)	13(11.30)		
Very difficulty	87(6.48)	85(7.40)	2(1.74)		
**Proactive health behaviors score**	0.70(0.39,1.09)	0.65(0.35,1.01)	1.48(1.01,2.05)	-11.077	＜0.001

Supplementary Table S3 The group-based trajectory model index fitting

**Fit statistics**	**Number of class**				
	**1**	**2**	**3**	**4**	**5**
AIC	-7542.03	-6862.25	-6741.11	-6654.78	-6613.45
BIC	-7538.81	-6880.09	-6733.06	-6688.59	-6652.47
aBIC	-7542.03	-6880.46	-6707.05	-6699.06	-6664.54
Entropy		0.946	0.880	0.871	0.875
**Class proportion, %**					

Class1	100	91.44	82.65	80.27	79.15
Class2		8.56	14.82	15.49	15.41
Class3			2.53	2.83	2.16
Class4				1.41	2.61
Class5					0.67
**Average posterior probabilities**					
Class1		0.98	0.96	0.95	0.94
Class2		0.94	0.85	0.83	0.79
Class3			0.94	0.86	0.80
				0.92	0.93
					0.99

Note: Continuous variables not normally distributed were described by M(P25, P75); Total : n=1343, 100.00%, Declining Group : n=1228, 91.44%,Improving Group : n=115, 8.56%.

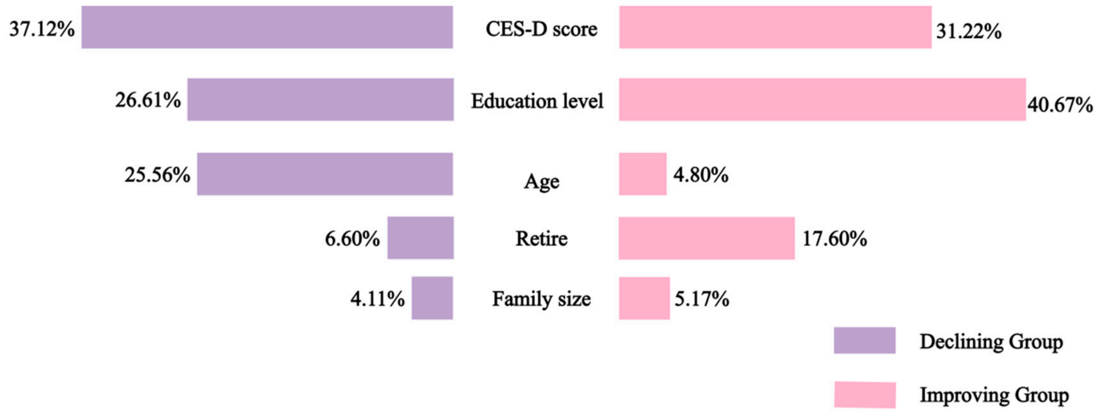

Supplementary Figure S2 Based on Shapley value decomposition, the contribution of mu ltiple factors of proactive health behavior trajectory in Chinese middle-aged and elderly p atients with multimorbidity

Supplementary Table S4 Latent class analysis model fitting index

Class	AIC	BIC	aBIC	Entrony	LMR(p)	BLRT(p)	Class proportions
1	14719.865	14792.702	14748.23	-	-	-	1.00
2	14393.943	14544.820	14452.699	0.501	<0.001	<0.001	0.52/0.48
3	14197.173	14426.090	14286.321	0.637	<0.001	<0.001	0.56/0.28/0.17
4	14107.946	14414.903	14227.486	0.676	0.025	<0.001	0.38/0.12/0.34/0.15
5	14054.315	14439.312	14204.247	0.681	0.003	<0.001	0.20/0.18/0.39/0.12/0.12
6	14029.574	14492.610	14209.896	0.718	0.095	<0.001	0.01/0.13/0.39/0.22/0.12/0.12

The evaluation indicators of LCA: The smaller the values of AIC, BIC, and aBIC, the better the model fit. The larger the ent ropy value, the more precise the classification. The likelihood ratio test index (LMR) and the bootstrap likelihood ratio test (B LRT) index are used to compare the fit differences of latent class models. When these two values reach a significant statistica l level ($P < 0.05$), it indicates that the model with K (the number of freely estimated parameters) classes is significantly bette r than the model with K-1 classes.

Supplementary Table S5 Probability distribution of five potential categories.

	**class1**	**class2**	**class3**	**class4**	**class5**	**Total**
**Hypertension**	71(9.89%)	45(18.99%)	400(77.07%)	43(25.75%)	157(100.00%)	671(49.96%)
**Diabetes**	17(4.18%)	6(2.53%)	138(26.59%)	6(3.59%)	0(0.00%)	161(1.19%)
**Cancer**	4(1.52%)	0(0.00%)	10(1.93%)	2(1.20%)	0(0.00%)	16(1.19%)
**Chronic lung diseases**	22(1.52%)	18(7.59%)	20(3.85%)	160(95.81%)	24(15.29%)	273(20.33%)
**Heart disease**	51(19.39%)	0(0.00%)	222(42.77%)	30(3.59%)	5(3.18%)	380(28.29%)
**Stroke**	20(7.60%)	0(0.00%)	36(6.94%)	2(1.20%)	9(5.73%)	67(4.99%)
**Mental illness**	20(7.60%)	0(0.00%)	2(0.39%)	0(0.00%)	1(0.64%)	23(1.71%)
**Arthritis**	442(77.95%)	237(100.00%)	204(39.31%)	67(40.12%)	157(100.00%)	870(64.78%)
**Dyslipidemia**	11(4.18%)	0(0.00%)	287（55.30%）	14(8.38%)	0(0.00%)	312(23.23%)
**Liver disease**	55(15.21%)	15(6.33%)	31（5.97%）	14(8.38%)	0(0.00%)	100(7.53%)
**Kidney disease**	122(46.39)	0(0.00%)	31（5.97%）	1（0.60%）	9(5.73%)	163(12.14%)
**Stomach disease**	386(56.39%)	237(100.00%)	180（34.68%）	58（34.73%）	0(0.00%)	624(46.46%)
**Asthma**	27(10.27%)	0(0.00%)	18（3.47%）	88（52.69%）	0(0.00%)	133(9.90%)
**Memory disorders**	18(5.32%)	4(1.69%)	15（2.89%）	0(0.00%)	2(1.27%)	35(2.61%)
**Total**	20(19.58%)	237(17.65%)	519(38.64%)	167(12.43%)	157(11.69%)	1343（100%）
